# Factors associated with mortality in hospitalised, non-severe, older COVID-19 patients – the role of sarcopenia and frailty assessment

**DOI:** 10.1186/s12877-022-03571-w

**Published:** 2022-12-07

**Authors:** Karolina Piotrowicz, Monika Ryś, Ian Perera, Barbara Gryglewska, Małgorzata Fedyk-Łukasik, Jean-Pierre Michel, Barbara Wizner, Wojciech Sydor, Agnieszka Olszanecka, Tomasz Grodzicki, Jerzy Gąsowski

**Affiliations:** 1 Department of Internal Medicine and Gerontology, Jagiellonian University Medical College, Kraków, Poland; 2 Department of Internal Medicine and Geriatrics, University Hospital in Kraków, Kraków, Poland; 3 University of Geneva, Geneva, Switzerland; 4 Center for Innovative Therapies, Clinical Research Coordination Center, University Hospital in Kraków, Kraków, Poland; 5 Department of Rheumatology and Immunology, Jagiellonian University Medical College, Kraków, Poland; 6 Department of Cardiology, Interventional Electrocardiology and Hypertension, Jagiellonian University Medical College, Kraków, Poland; 7 Department of Internal Medicine and Gerontology, Jagiellonian University Medical College, University Hospital, 2 Jakubowskiego St., building I, 5th floor, 30-688 Kraków, Poland

**Keywords:** COVID-19, Skeletal muscles, Sarcopenia, Frailty, Handgrip strength, Dynapenia, SARC-F, Rockwood

## Abstract

**Background:**

COVID-19 has affected older persons the most. The propensity to have severe COVID-19 or die of the infection was especially prevalent among older subjects with multimorbidity, frailty and sarcopenia. The aim of our study was to check which of the simple clinical biomarkers, including the assessment of muscle and frailty, would associate with the survival and the length of hospital stay in older patients with COVID-19. An additional aim was to report the influence of chronic diseases, chronic medication use, and COVID-19 signs and symptoms on the aforementioned outcome measures.

**Methods:**

The CRACoV study was a prospective single-center (University Hospital in Krakow, Krakow, Poland) observational study of clinical outcomes in symptomatic COVID-19 patients that required hospital treatment. We analysed data of persons aged ≥ 65 years. We assessed muscular parameters in accordance with EWGSOP2, frailty with the Rockwood Clinical Frailty Scale. We used the data of the initial and 3-month assessment. Demographic characteristics, past medical history, and baseline laboratory values were gathered as a part of routine care. We calculated sex and age, and additionally number-of-diseases adjusted odds ratios of mortality associated with studied factors and betas of the relation with these factors and the length of hospital stay.

**Results:**

The mean (standard deviation, SD) age of 163 participants (44.8% women, 14.8% died) was 71.8 (5.6) years, age range 65–89 years. One score greater SARC-F was associated with 34% (*p* = 0.003) greater risk of death, and 16.8 h longer hospital stay (*p* = 0.01). One score greater Rockwood was associated with 86% (*p* = 0.002) greater risk of death, but was unrelated to the length of hospital stay. Hand grip strength and dynapenia were unrelated to mortality, but dynapenia was related to longer hospital stay. Probable sarcopenia was associated with 441% (*p* = 0.01) greater risk of death.

**Conclusions:**

In conclusion, the patient assessment with SARC-F and the Rockwood Clinical Frailty Scale may significantly improve the prediction of outcomes in older patients with COVID-19 and by extension might be of use in other acute severe infections. This, however, requires further research to confirm.

## Introduction

COVID-19 has affected older persons the most. The propensity to have severe COVID-19 or die of the infection was especially prevalent among older subjects with multimorbidity, frailty and sarcopenia [1, 2]. What these persons have in common is the narrowed ability to adequately compensate for the changes in the internal and external environment, higher degree of baseline age-related inflammation usually referred to as ‘inflammageing’, and higher overall risk of adverse health-related outcomes including high risk of cardiovascular events [3, 4].

Skeletal muscle undergoes a profound change with ageing. When pathologically advanced, this change may assume a form of dynapenia, namely low muscle strength [5]. When it couples with low muscle mass we diagnose sarcopenia, as indicated in the European Working Group on Sarcopenia in Older People revised guidelines (EWGSOP2) [6]. Sarcopenia as an age-related loss of muscle mass and function is an important element of physical frailty [7]. The SARS-CoV-2 infection with severe inflammation and high catabolism may have a great impact on the structure and function of skeletal muscle [8]. Such acute sarcopenia may adversely influence the course of the infection in the older patient, thus adding to the already high burden of the disease. Some data suggest that sarcopenia at the time of hospital admission may influence the length of hospital stay or may increase mortality in patients with moderate to severe COVID-19 [9, 10].

There are several tools to assess muscle mass, strength, and performance [5]. Some of these can be used at the bedside and have been shown to be related to all-cause mortality and outcome in chronic and acute diseases, including COVID-19 [2, 11].

There is still a relative paucity of data concerning the acute change, and its direction (i.e. increase or decrease), of muscle mass and strength in hospitalized older patients with COVID-19. Likewise, relatively little is known about the relation between simple clinical measures of sarcopenia, dynapenia and muscle strength on one hand and mortality and length of hospital stay due to COVID-19 on the other, especially, in the context of diseases, medications, and symptoms of COVID-19. Still less data are available pertaining to relatively healthy older persons, with good overall functional status. This may be of special importance as deaths from COVID-19, or the development of the post-COVID-19 syndrome, occurred also in young and apparently healthy and fit persons [12].

The aim of our study was to check which of the biomarkers, including simple clinical biomarkers for the assessment of muscle-related health, frailty, chronic diseases, chronic medication use, and COVID-19 signs and symptoms would be associated with risk of poor survival and the increased length of hospital stay in older patients with COVID-19.

## Materials and methods

We analysed data of persons aged ≥ 65 years enrolled in the multidisciplinary study of SARS-CoV-2 infection in a population of adults hospitalized due to COVID-19 infection (CRACoV-HHS: CRAcow in CoVid pandemic — Home, Hospital and Staff). The design of the CRACoV study, a prospective single-center (University Hospital in Krakow, Krakow, Poland) observational study of clinical outcomes of symptomatic COVID-19 patients, has been described in detail elsewhere [13]. Briefly, patients 65 years of age or older who were hospitalized from January 2021 to June 2021, in dedicated COVID-19 wards of the University Hospital in Krakow were invited to participate in a geriatric sub-study of the project. We arbitrarily assumed that the difference of 4 kg in the hand grip strength would be clinically relevant and reproducible change. We found that a sample of 100 persons would be enough to detect a clinically relevant difference in the hand grip strength by standardised difference of 0.5 with the power 80% and the standardised difference of 0.7 with the power of 95%, and that 200 patients would be required to detect the standardised mean difference of 0.45 with the power of 90% and the standardised mean difference of 0.4 with the power of 80%, all at 5% significance level. The protocol of the study provided for the inclusion of 180 patients. Such sample, assuming the possible 30% discontinuation rate, should be enough to meet the predefined sample-size criteria. We included all consecutive patients meeting the age criterion who at the time of admission did not require intensive care. The exclusion criterion was the inability to sign informed consent to participate. The participants were consecutively assessed by certified physiotherapists or geriatricians. The assessment was performed on hospital admission and at a 3-month follow-up outpatient visit. A simplified screening assessment for sarcopenia (SARC-F, an evaluation battery of 5 questions), and frailty syndrome (Rockwood Clinical Frailty Scale, a descriptive evaluation battery with 9 levels of functionality which is based on assessment of disease, physical activity, and dependence) has been performed with the use of validated tools [14, 15]. Sarcopenia and dynapenia were diagnosed according to the EWGSOP2 guidelines [6]. Muscle strength was measured during each assessment session, three times for each hand in a standard sitting position with a handheld dynamometer (Jamar®5030J1). The highest value of muscle strength of the dominant hand was used for the subsequent analyses. Low muscle strength (dynapenia) was diagnosed if the muscle strength was lower than 16 kg for women and 27 kg for men, respectively. According to the EWGSOP2 algorithm, probable sarcopenia was diagnosed in patients with SARC-F ≥ 4 points with coexisting dynapenia [6]. Calf circumference of the dominant leg was used as a proxy measure for skeletal muscle mass. We measured body weight and height and calculated body mass index (kg/m^2^). We collected information concerning the signs and symptoms of COVID-19, the data enabling the disease severity assessment according to the National Institutes of Health (NIH) classification (into asymptomatic, mild, moderate, severe, and critical), and chronic medications and comorbidities [16]. Demographic characteristics and baseline laboratory values were gathered as part of routine care. The blood oxygen saturation (SpO_2_) was measured using finger pulseoximeter. Tachypnea was defined as a respiratory rate of ≥ 20/min. The length of hospital stay was time from admission to emergency department to final hospital discharge. The survival was ascertained at the time of the follow-up outpatient visit after three months post discharge. In the case of patients who did not attend, vital status was assessed using the information embedded in the hospital electronic health records, and over the telephone with the proxy indicated in the medical documentation.

As a part of a history, we obtained information on any new sign or symptom that was associated with the COVID-19 infection that was the cause for hospitalisation. We also obtained an information concerning medical history and current medications from 14 pre-specified classes, and further corroborated with medical documentation as supplied by the patient’s family, including the list of current medications. In the case of patients previously hospitalised in our hospital we checked the previous diagnoses and the recent prescription. The list of chronic conditions and groups of conditions reported on admission included: depression, immune diseases, liver diseases, cancer (past and active), thyroid disease, chronic kidney disease, deep vein thrombosis, peripheral arterial disease, diabetes mellitus, hypertension, previous stroke, previous myocardial infarction, atrial fibrillation, heart failure, coronary heart disease, chronic obstructive pulmonary disease and asthma.

As part of standard hospital procedures blood samples were obtained at the baseline for the standard assessment of c-reactive protein (CRP) and interleukin 6 (IL-6) assays.

The study was approved by the Bioethics Committee of Jagiellonian University Medical College in Krakow, Poland (number: 1072.6120.333.2020). All participants signed informed consent to take part.

### Statistical analysis

Data management and analyses were performed with SAS 9.4 (SAS Institute Inc., Cary, NC, USA). Continuous variables were compared with a standard normal Z test of Wilcoxon’s test as appropriate; proportions were compared with a chi-square test. Ordinal descriptive variables such as Rockwood Clinical Frailty Scale and the disease severity assessment according to the National Institutes of Health (NIH) classification, were recoded into linear numerical ordinal variables. We modelled the probability of death using logistic regression models with the adjustment for sex and age, and the additional adjustment for number of diseases present, or, in the case of medications, for the potential indications. We also fitted similarly adjusted models with the length of hospital stay as a dependent variable. In the final step of our regression analyses we re-run all logistic regression models 2 with additional stepwise adjustment for factors that in our analyses turned out to be significantly associated with fatal outcome. We adopted a 5% two-sided value for statistical significance.

## Results

### The groups characteristics

The mean (standard deviation, SD) age of 163 participants (44.8% women) was 71.8 (5.6) years, age range 65–89 years. Twenty-four (14.7%) died. Patients were on average (SD) burdened with 2.9 (2.2) diseases, ranging 0–14. Multimorbidity was present in 68.5% and was significantly more frequent in persons who died compared with survivors (91.7% vs. 64.5%, *p* = 0.008). Compared with those who survived 3 months, patients who died had greater median (5–95 percentile) SARC-F (2 [0–9] vs. 1 [0–7] *p* = 0.007), greater Rockwood Clinical Frailty Scale (2.5 [0–9] vs. 2 [0–4], *p* = 0.04), and greater number of diseases (4 [1-9] vs. 2 [0–7], *p* < 0.0001). The mean BMI was 29.1 (5.1) kg/m^2^, the calf circumference was 36.8 (4.3) cm, and the hand grip strength was 23.3 (10.8) kg [16.4 (7.0) and 28.9 (10.1) kg for women and men respectively, [*p* < 0.0001]. There were no differences in initial median pO2 (91 [80–100]%). Among survivors, by the 3-month follow-up visit, hand grip strength increased by 5.3 (6.0) kg in women, and 9.2 (8.7) kg in men (p for change and between gender comparison < 0.0001). Dynapenia was present in 40.3% participants, and its frequency did not significantly differ between those who died and survived (56.5% vs. 37.5%, *p* = 0.09). At baseline patients on average (SD) had a SARC-F score of 1.9 (2.5), range 0–10. Based on SARC-F 22.2%, and based on SARC-F combined with dynapenia 14.7%, patients had suspected, and probable sarcopenia, respectively. The corresponding values for Rockwood Clinical Frailty Scale was 2.0 (1.3), range 0–6. Based on Rockwood Clinical Frailty Scale 29.6% of patients were pre-frail or frail. The baseline severity of the disease (NIH) was 2.2 (0.9) and it did not differ between survivors and non-survivors. The median (5th-95th percentile) admission C-reactive protein (CRP) in patients who have died was 93.1 (2.0-221.0) mg/l and was higher than in survivors (53.6 [6.5–169.0] mg/l, *p* = 0.02). Admission interleukin 6 (IL-6) was 55.8 (7.6–1781.0) pg/ml in patients who died and was greater than in survivors (25.6 [2.5-135.3] pg/ml, *p* = 0.04). The mean (SD) length of hospital stay was 16.1 (8.3) days The frequencies of particular diseases, signs and symptoms, and medications are contained in Table 1.

**Table 1:** The percentages of chronic diseases, signs and symptoms and chronic medications in the entire group and in survivors and non-survivors

	Entire group (***n***=163)	Survivors (***n***=139)	Non-survivors (***n***=24)	***p*** value*
**Diseases (% affected)**				
Depression	5.6	5.1	8.3	0.53
Immune disease	1.2	0.7	4.2	0.16
Liver disease	6.9	6.7	8.3	0.77
Cancer past	7.5	6.6	12.5	0.71
Cancer current	6.2	4.4	16.7	0.02
Thyroid disease	17.9	16.7	25.0	0.33
Chronic kidney disease	6.3	3.7	20.8	0.001
Venous thromboembolism	5.6	5.8	4.2	0.74
Peripheral arterial disease	10.1	9.0	16.7	0.25
Diabetes mellitus	35.4	30.7	62.5	0.003
Hypertension	82.6	81.9	87.0	0.55
Stroke	5.6	3.7	17.4	0.008
Atrial fibrillation	18.8	16.2	33.3	0.05
Heart failure	12.2	9.0	31.8	0.002
Myocardial infarction	17.1	12.6	43.5	0.0003
Coronary heart diseases	29.0	25.0	52.2	0.008
Chronic obstructive pulmonary disease	9.3	8.0	16.7	0.18
Asthma	9.3	8.0	16.7	0.18
**Signs and symptoms (% affected)**				
Tachypnea	16.9	13.7	22.7	0.01
Oedema	5.8	5.4	8.7	0.52
Diarhorrea	31.2	31.8	27.3	0.67
Nausea/vomiting	17.5	17.4	18.2	0.93
Chest pain	18.2	18.2	18.2	1.00
Skin leasions	3.3	3.0	4.6	0.71
Back pain	34.4	36.4	22.7	0.22
Muscular pain	44.8	47.7	27.3	0.07
Malaise	86.4	87.1	81.8	0.50
Headache	37.0	37.9	31.8	0.59
Running nose	16.9	16.7	18.2	0.86
Sore throat	19.5	17.4	31.8	0.12
Ageusia	16.9	15.9	22.7	0.43
Anosmia	16.2	15.2	22.7	0.37
Dyspnoe	74.0	72.0	86.4	0.15
Cough	70.8	72.0	63.6	0.43
Shivers	44.8	48.5	22.7	0.02
Fever	75.3	75.8	72.7	0.76
Subfebrile	58.4	60.6	45.5	0.18
Asymptomatic	6.0	5.5	9.5	0.47
**Medications (% taking)**				
Vitamin D	24.4	25.4	18.2	0.47
Proton-pump inhibitors	19.9	18.8	26.1	0.42
Inhaled non-steroid drugs	14.3	13.8	17.4	0.65
Inhaled steroid drugs	11.1	9.4	20.8	0.10
Non-steroidal anti-inflammatory drugs	28.3	28.7	26.1	0.80
Antidepressants	7.4	5.8	16.7	0.06
Neuroleptics	1.2	0.7	4.4	0.15
Insulin	9.3	6.5	26.1	0.003
Sulphonylureas	11.9	11.0	17.4	0.38
Flosin	1.3	0.7	4.4	0.15
Metformin	26.9	23.4	47.8	0.01
Non-vitamin K antagonist oral anticoagulants	11.1	9.4	20.8	0.10
Vitamin K antagonists	5.0	3.7	12.5	0.07
Aspirin	29.0	25.4	50.0	0.01
Statins	40.3	39.0	47.8	0.42
Alfa-blockers	14.7	13.4	21.7	0.30
Mineralocorticoid receptor antagonists	6.4	4.5	17.4	0.02
Calcium channel blockers	28.0	29.1	21.7	0.47
Diuretics	46.5	44.8	56.5	0.30
Beta-blockers	59.9	56.7	78.3	0.05
Angiotensin receptor blocker	18.5	18.7	17.4	0.89
Angiotensin-converting enzyme inhibitors	45.5	44.4	52.2	0.49

* For difference between survivours and non-survivours

### Mortality and length of stay as function of measures-of-muscle

Of the included patients 14.8% died. SARC-F (*p* = 0.003), and Rockwood Clinical Frailty Scale (*p* = 0.002), in separate logistic regression models, each time with adjustment for sex and age, were related to mortality (Table 2). In analogous linear regression models, length of stay was related to dynapenia (*p* = 0.03), calf circumference (*p* = 0.01), and SARC-F (*p* = 0.01). One score greater SARC-F was associated with 34% (*p* = 0.003) greater risk of death, and 16.8 h longer hospital stay (*p* = 0.01). One score greater Rockwood was associated with 86% (*p* = 0.002) greater risk of death, but was unrelated to the length of hospital stay. After additional adjustment for number of diseases, mortality was related to SARC-F (*p* < 0.05), and Rockwood Clinical Frailty Scale (*p* = 0.05). After additional adjustment for the number of diseases present, none of the measures-of-muscle were related to the length of stay (Table 2). The hand grip strength measured at the beginning of hospitalisation did not influence mortality or length of hospital stay. The probable sarcopenia was significantly associated with worse survival in both models (both *p* ≤ 0.03) but was not related to the length of hospital stay. Probable sarcopenia was associated with 441% (*p* = 0.01) greater risk of death. After additional adjustments (stepwise selection optimised models 2s) probable sarcopenia (OR 6.25 [1.24–31.49], *p* = 0.03) retained its significance in prediction of fatal events. Of note, tachypnoea (OR 1.28 [95%CI 1.06–1.54], *p* = 0.002), number of diseases (OR 1.49 [1.16–1.91], *p* = 0.009) and active cancer (OR 9.10 [1.27–65.2], *p* = 0.03) were significantly associated with the risk of death. When CRP and IL-6 were additionally offered in the stepwise procedure, probable sarcopenia lost its significance. In this model CRP was a significant predictor of fatal outcome (OR 1.01 [1.00- 1.03], *p* = 0.02).

**Table 2:** The odds ratios of mortality associated with studied factors, and beta values for relation with the studied factors and length of hospital stay

	SpO_**2**_	SARC_F	Rockwood	Handgrip	Dynapenia (0/1)	Sarcopenia probable (0/1)	Calf circumference	BMI	Number of diseases	Multimorbidity (0/1)
**Model 1:** adjusted for age and sex										
**Mortality**	0.98 (0.91-1.06) *p*=0.64	1.34 (1.11-1.62) *p*=0.003	1.86 (1.25-1.77) *p*=0.002	0.98 (0.94-1.03) *p*=0.52	1.91 (0.75-4.87) *p*=0.17	4.41 (1.41-13.80) *p*=0.01	0.96 (0.85-1.08) *p*=0.51	1.03 (0.94-1.13) *p*=0.50	1.58 (1.27-1.97) *p*<0.0001	6.28 (1.39-28.44) *p*=0.02
**LoS beta(SEM)**	-0.45 (0.14) *p*=0.001	0.76 (0.29) *p*=0.01	0.42 (0.54) *p*=0.44	-0.10 (0.07) *p*=0.20	2.79 (1.27) *p*=0.03	3.10 (1.92) *p*=0.11	0.36 (0.14) *p*=0.01	0.11 (0.13) *p*=0.41	1.13 (0.32) *p*=0.0005	2.99 (1.40) *P*=0.03
**Model 2:** adjusted for age, sex and number of diseases										
**Mortality**	1.01 (0.93-1.10) *p*=0.82	1.23 (1.0-1.52) *P*<0.05	1.55 (1.0-2.39) *p*=0.05	1.0 (0.95-1.05) *p*=0.85	1.37 (0.49-3.81) *p*=0.55	3.92 (1.15-13.41) *p*=0.03	0.93 (0.84-1.03) *p*=0.18	1.0 (0.91-1.10) *p*=0.96		
**LoS beta(SEM)**	-0.37 (0.14) *p*=0.009	0.53 (0.29) *p*=0.07	-0.13 (0.55) *p*=0.81	-0.08 (0.07) *p*=0.26	2.05 (1.25) *p*=0.11	1.90 (1.79) *p*=0.29	0.20 (0.15) *p*=0.19	-0.4 (0.13) *p*=0.77		

Multimorbidity: ≥2 diseases present; *SpO2* blood oxygen saturation, *BMI* body mass index (kg/m^2^), *LoS* length of stay, *SEM* standard error of the mean

### Mortality and length of hospital stay as functions of diseases, signs and symptoms, and medications

**Fig. 1:**
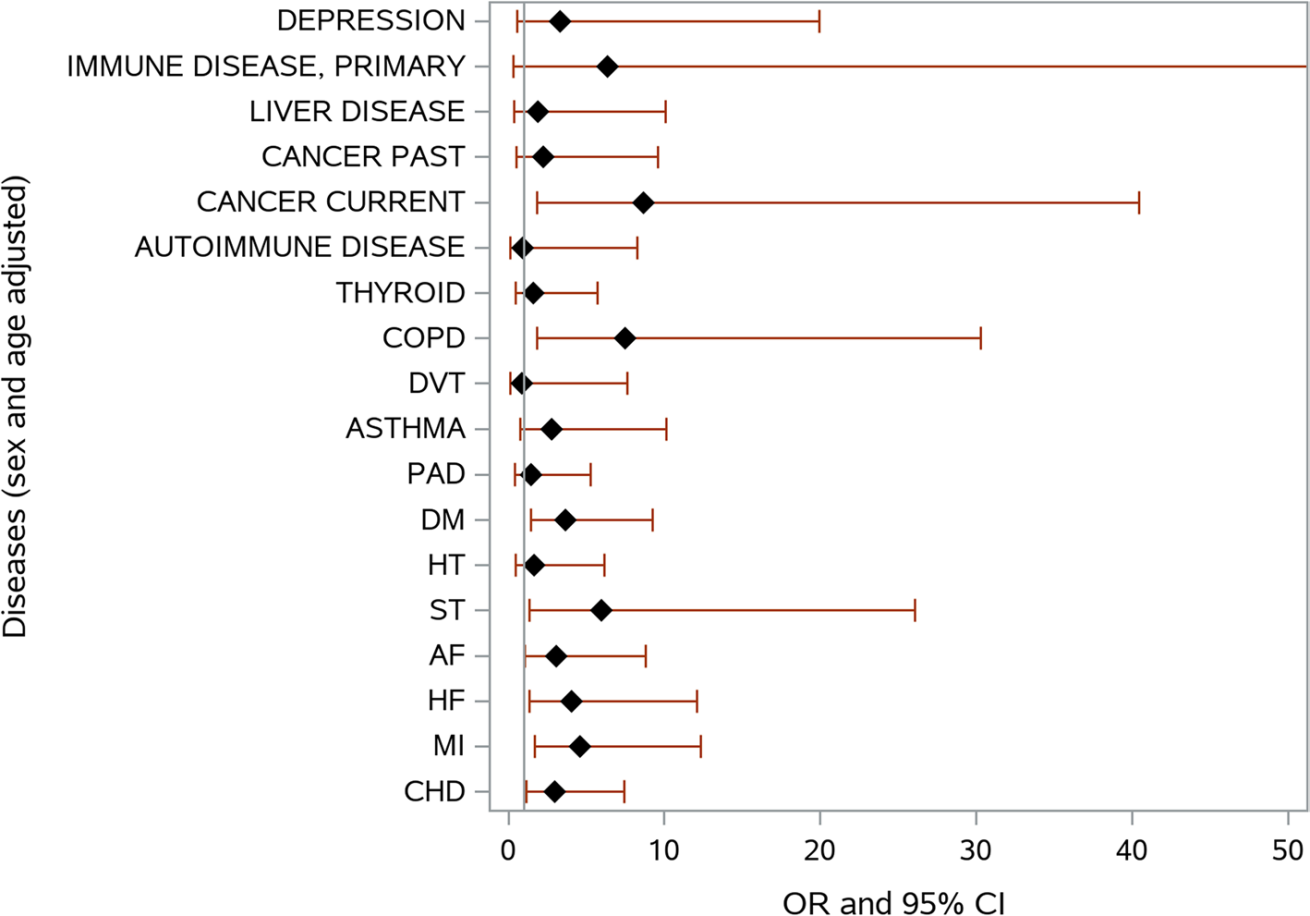
Risk of death during COVID-19 infection as a function of chronic diseases. Adjusted for sex and age. *OR, odds ratio, 95% CI, 95% confidence interval; DEPR, depression; IMMUNE, immune disease; LIVER, liver disease; THYR, thyroid disease; CKD, chronic kidney disease; DVT, venous thromboembolism; PAD, peripheral arterial disease; DM, diabetes mellitus; HT, hypertension; ST, stroke; AF, atrial fibrillation; HF, heart failure; MI, myocardial infarction; CHD, coronary heart disease; COPD, chronic obstructive pulmonary disease

**Fig. 2:**
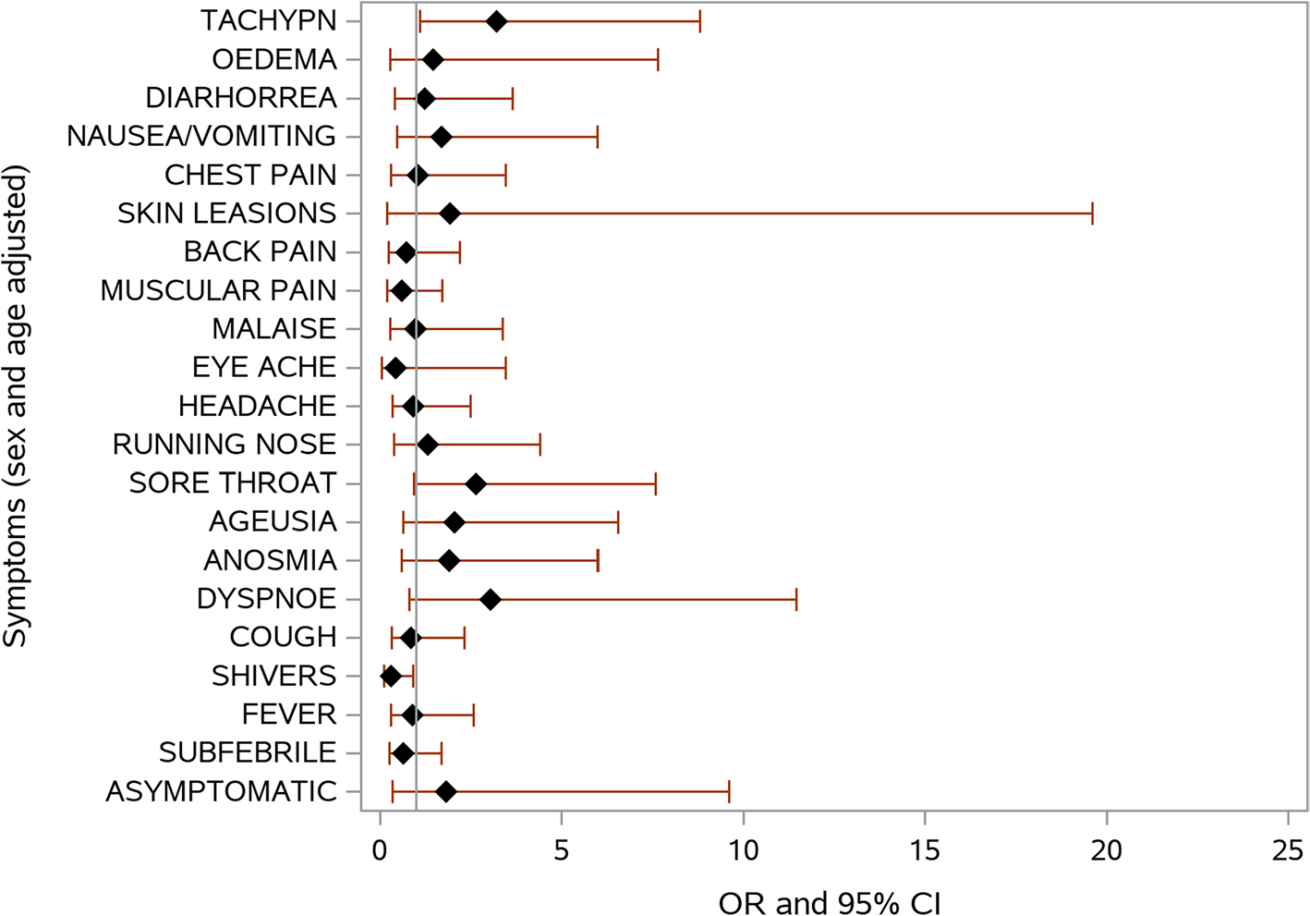
Risk of death during COVID-19 infection as a function of signs and symptoms. Adjusted for sex and age. *OR, odds ratio, 95% CI, 95% confidence interval; TACHYPN, tachypnea

**Fig. 3:**
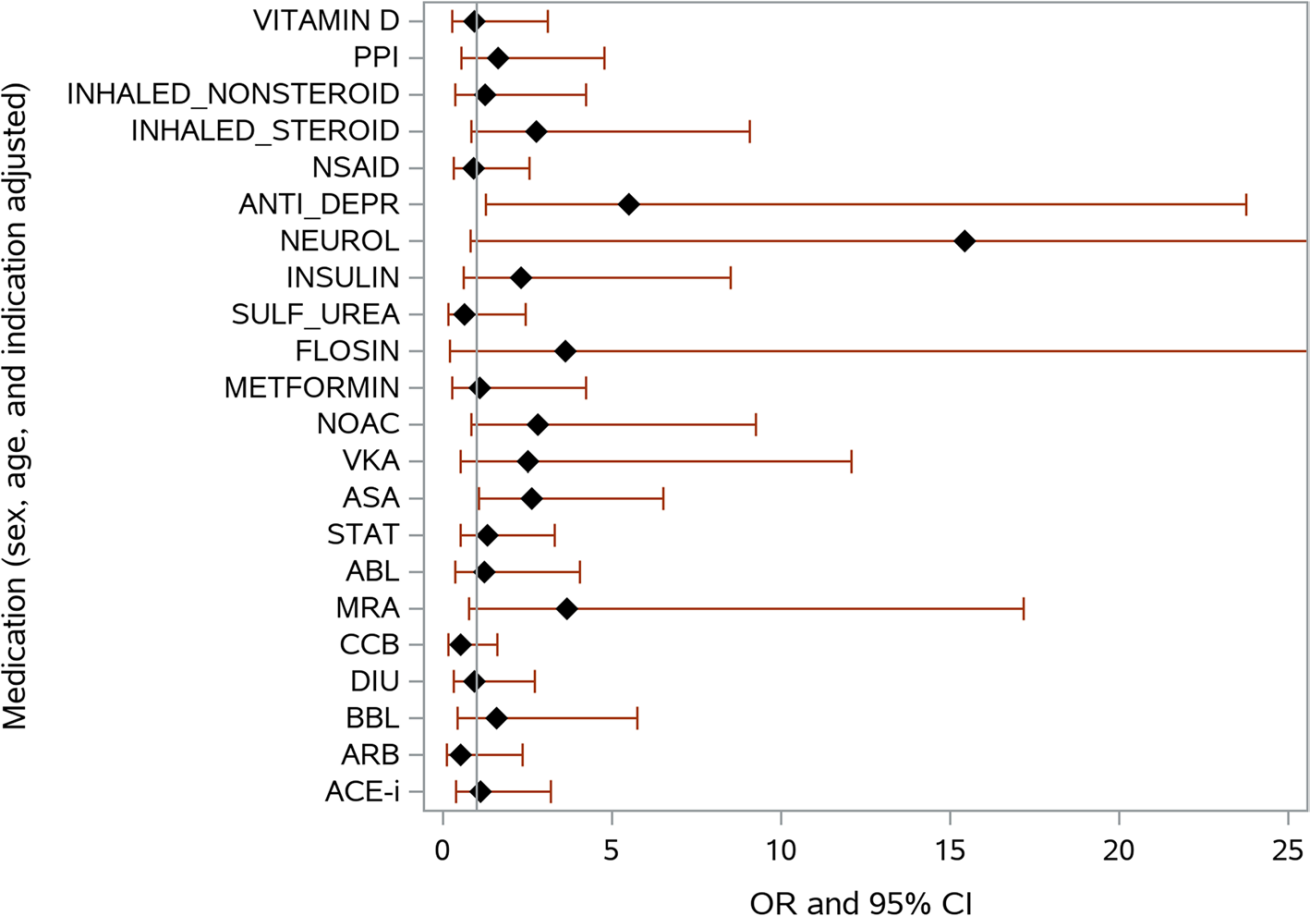
Risk of death during COVID-19 infection as a function of chronic medications. Adjusted for sex and age. *OR, odds ratio, 95% CI, 95% confidence interval; PPI, proton-pump inhibitors; NSAID, non-steroidal anti-inflammatory drugs; ANTI_DEP, antidepressants; NEUROL, neuroleptics; SULF_UREA, sulphonylureas; NOAC, non-vitamin K antagonist oral anticoagulants; VKA, vitamin K antagonists; ASA, aspirin; STAT, statins; ABL, alfa-blockers; MRA, mineralocorticoid receptor antagonists; CCB, calcium channel blockers; DIU, diuretics; BBL, beta-blockers; ARB, Angiotensin receptor blocker; ACE-I, angiotensin-converting enzyme inhibitors

Greater number of chronic diseases, and in a separate model multimorbidity, with adjustment for sex and age, were associated with higher risk of death (both *p* ≤ 0.02). They were also associated with longer length of hospital stay (both *p* ≤ 0.03) (Table 2). Of the diseases, current cancer, chronic obstructive pulmonary disease, chronic kidney disease, previous stroke, atrial fibrillation, heart failure, previous myocardial infarction, and coronary artery disease adversely affected the chance of survival (all *p* ≤ 0.05) (Fig. 1). Of signs and symptoms listed, tachypnea were associated with risk of worse survival (Fig. 2). Initial SpO_2_ was not related to the risk of death but was inversely related to the length of hospital stay (*p* ≤ 0.009) (Table 2). Of medications, previous use of aspirin or antidepressants was associated with greater risk of death (both *p* ≤ 0.05) (Fig. 3).

## Discussion

The severe acute respiratory syndrome due to coronavirus infection has been linked to the loss of muscle mass and strength [2]. The pathophysiologic background for these associations ranges from inactivity, low food intake, through low blood oxygen saturation to inflammation, which all impact muscle energy metabolism [8, 11]. In our analyses there was no relation between hand grip strength or dynapenia and the outcomes we studied. We hypothesize that an acutely decreased muscle strength may reflect changes resulting from generally worsened condition due to inflammation, fever or dyspnoea, and not necessarily acute dynapenia. Also, the setting of a COVID-19 ward, with staff wearing personal protective equipment, and the anxiety experienced by patients may have influenced the performance of the hand grip strength assessment. On the other hand, SARC-F assessed on admission, which reflected the two-week period prior to hospitalisation, has been associated with the outcome. This is in line with the report by Riesgo et al. who found a SARC-F of ≥ 4 to be related to mortality [17]. However, their group was older (86.1 [8.7] years), and relatively healthier than ours. In their group, persons with SARC-F ≥ 4 predominated. Nevertheless, their hospital stay was shorter than that of our patients. Riesgo et al. did not adjust their models for concomitant conditions or multimorbidity [17].

The fact that in our analyses the SARC-F or Rockwood Clinical Frailty Scale seem to have performed better than a direct measure of muscle strength may stem from several factors. First, the information in SARC-F reflected a steady state that preceded the infection and might thus have been a better descriptor of sarcopenia at the moment of hospital admission. Second, SARC-F is influenced by more factors than hand grip strength. It comprehensively assesses both upper and lower extremities, and to a certain degree the overall ability for independent mobility [14, 18]. In a situation of the COVID-19 ward with only limited rehabilitation, SARC-F might predict the ability of the patient to ambulate.

We found that in a group of older adults hospitalised due to COVID-19 infection, after three months hand grip strength improved in the survivors. This may reflect the degree to which acute COVID-19 infection may have caused acute dynapenia in the first instance. This is in line with the 12-month observation of a cohort of COVID-19 patients from Belgium [19]. A 6-month observation of a French cohort revealed the reversibility of sarcopenia [20].

Cheval et al., based on data of 3600 persons from the Survey of Health, Ageing and Retirement in Europe (SHARE) cohort, reported that higher hand grip strength assessed between 2004 and 2017 was associated with lower risk of hospitalisation due to COVID-19 in 2020 [21]. It may be postulated that the hand grip assessment in that study reflected the steady state of the muscles, unaffected by acute disease. In our report we concentrate on the patient status on admission and after three months in those who survived. Del Brutto et al. assessed the hand grip strength in 254 COVID-19-free older individuals with mean (SD) age of 70.2 (7.7) years, who were then followed-up for possible SARS-CoV-2 seroconversion. In this study the risk of 5% decline in hand grip strength was more probable in participants who seroconverted [22].

Several other reports addressed the issue of muscle mass, strength or function in relation to the outcome of COVID-19 [23–25]. We found, based on the definitions from EWGSOP2, that probable sarcopenia predicted mortality in older COVID-19 patients. Meyer et al. performed a meta-analysis of the studies that addressed the relation between lean skeletal muscle mass (LSMM) and mortality or length of hospital stay. They based their assessment of muscle mass on summary data of 1059 computed tomography images of patients aged 48–66 years. While in the univariate analysis LSMM was significantly predicting mortality, the relation lost significance after adjustment, however the factors for which the model was adjusted were not reported [26]. Of note, the finding by Meyer et al. is in line with the findings in other severe acute conditions such as sepsis, trauma, surgery, or severe oncologic conditions [27].

Greater number of diseases and presence of multimorbidity were related to mortality and longer hospital stay. Of the single diseases, chronic obstructive pulmonary disease, current cancer, and diseases related to risk of atherosclerosis (diabetes mellitus) or being a consequence of it (previous stroke, previous myocardial infarction, coronary artery disease, atrial fibrillation, heart failure), and chronic kidney disease were related to higher mortality.

Casas-Deza et al. reported that the presence of any comorbidities in older hospitalised patients with COVID-19 has been associated with greater mortality [28]. In a study by Junior et al., cancer, and chronic atrial fibrillation were related to higher mortality [29]. Several studies showed a relation between chronic kidney disease and the risk of mortality [30, 31]. This was confirmed in a broader range of diseases in other studies [32–34].

Other published data indicate that three or more diseases and a Charlson index of ≥ 3 are related to greater mortality in older COVID-19 patients [35, 36]. This is in line with our results. The relation between the Charlson index and mortality is of special importance as it is in line with our results on SARC-F and the Rockwood Clinical Frailty Scale, all three being comprehensive tools enquiring into a broad spectrum of factors associated with functionality and disease burden in older patients. Similarly, a study by Assis et al. demonstrated a relation between the VES-13 result and adverse outcome after hospitalisation due to COVID-19, and a study by Fujita et al. reported the relation between worse admission ADL and greater hospital mortality due to COVID-19 [37, 38]. The association between frailty and severe COVID-19 infection was estimated among the UK Biobank participants hospitalized from COVID-19 using two frailty classifications: frailty phenotype and the frailty index. The study showed that frailty was associated with a higher risk of severe COVID-19 infection resulting in hospital admission or death, irrespective of sociodemographic and lifestyle factors and methods of frailty measurements [39]. The Clinical Frailty Scale by Rockwood et al. is one of the more often used frailty screening tools, also in COVID-19 patients [15, 40]. In a large population of the COVID-19 in Older PEople (COPE) study, disease outcomes were better predicted by the result of the Rockwood Clinical Frailty Scale than either age or comorbidity [41]. However, the prospective analysis of the UK Biobank data did not confirm the relationship between frailty and multimorbidity and adverse clinical outcome of COVID-19 [42]. Several studies that were further summarised in meta-analyses indicated that the Rockwood Clinical Frailty Scale is adversely related to survival of older patients with COVID-19 [40, 43]. Our results that the Rockwood Clinical Frailty Scale was significantly related to greater risk of death corroborate these findings.

Surprisingly, in our cohort, most of the signs and symptoms recorded on admission were not related to mortality or length of stay. The exception was tachypnea. This stands in contrast with some other studies which indicated that, productive cough or myalgias, and dyspnoea may among other symptoms be associated with worse prognosis [30, 44]. The same study indicated that SpO_2_ < 95% on room air predicted mortality [30]. In our cohort we found that lower SpO_2_ was related to longer hospital stay but not greater risk of mortality. The possible explanation is that our patients with lower SpO_2_ values had more severe disease that required longer therapy and monitoring.

In our cohort we found that aspirin and antidepressant use was related to greater mortality. Monserrat Villatoro et al. found that among eight studied medications, aspirin was one of those related to worse outcomes [45]. This is in contrast to data from the Veterans Health Administration that demonstrated lower risk of short-term COVID-19 mortality [46]. However, a meta-analysis of three studies showed that aspirin did not influence mortality [47]. The finding of the antidepressant influence on worse survival was not found in other published reports. Indeed, several reports demonstrated lower risk of intubation and mortality in COVID-19 patients who received SSRIs [48, 49]. Many of these studies were performed on younger populations, and in some reports the studied effect was that of SSRIs administered during hospitalisation [50, 51]. In our analysis no medication use prior to submission was related with improved survival. Notably, larger studies indicated that the pre-treatment with ACE-I or ARB would have beneficial effects in this regard [52]. Some studies indicated that polytherapy has been associated with mortality [44, 53]. However, due to the fact that the data on only prespecified 14 medications has been systematically gathered, we did not analyse this effect.

We found that by 3-month visit hand grip strength increased, which we believe to be a protective phenomenon. Previously, Chou et al. found that greater baseline hand grip strength was found to be associated with less cognitive decline after 10-year follow-up [54]. Hashida et al. found a positive association between hand grip strength and functional improvement after hip fracture [55].

Our study needs to be considered in the context of its limitations. First, our group consisted of a relatively small number of consecutive older patients from a larger project, of whom 24 persons died. Thus, although our analyses were prespecified, the sample-size calculation was performed for the effect on the change in hand grip strength. Consequently, the numbers may have been too low for other measures and this may have obscured some of the relations such as the influence of inflammatory markers on mortality. Likewise, some statistically significant results may have been driven by chance that arose from multiple testing. The fact that by default we did not include critically ill patients or severely demented patients means that the selection bias could not have been avoided. This needs to be taken into account when interpreting our results. Our cohort was examined between January and June 2021, and thus other published data gathered during different pandemic waves may have pertained to infections with SARS-CoV-2 variants of different virulence.

In conclusion, the patient assessment with SARC-F and the Rockwood Clinical Frailty Scale may significantly improve the prediction of outcomes in older patients with COVID-19 and by extension might be of use in other acute severe infections. This, however, requires further research to confirm.

## Data Availability

Upon reasonable request to the study Data Committee (jerzy.gasowski@uj.edu.pl, karolina.piotrowicz@uj.edu.pl).

## References

[1] Marengoni A, Zucchelli A, Vetrano DL, Armellini A, Botteri E, Nicosia F (2021). Beyond Chronological Age: Frailty and Multimorbidity Predict In-Hospital Mortality in Patients With Coronavirus Disease 2019. J Gerontol A Biol Sci Med Sci.

[2] Siahaan YMT, Hartoyo V, Hariyanto TI, Kurniawan A (2022). Coronavirus disease 2019 (Covid-19) outcomes in patients with sarcopenia: A meta-analysis and meta-regression. Clin Nutr ESPEN.

[3] Di Bari M, Tonarelli F, Balzi D, Giordano A, Ungar A, Baldasseroni S (2022). COVID-19, Vulnerability, and Long-Term Mortality in Hospitalized and Nonhospitalized Older Persons. J Am Med Dir Assoc.

[4] Piotrowicz K, Gąsowski J (2020). Risk Factors for Frailty and Cardiovascular Diseases: Are They the Same?. Adv Exp Med Biol.

[5] Cruz-Jentoft AJ, Sayer AA, Sarcopenia (2019). Lancet.

[6] Cruz-Jentoft AJ, Bahat G, Bauer J, Boirie Y, Bruyère O, Cederholm T (2019). Sarcopenia: revised European consensus on definition and diagnosis. Age Ageing.

[7] Cesari M, Landi F, Vellas B, Bernabei R, Marzetti E (2014). Sarcopenia and physical frailty: two sides of the same coin. Front Aging Neurosci.

[8] Piotrowicz K, Gąsowski J, Michel J-P, Veronese N (2021). Post-COVID-19 acute sarcopenia: physiopathology and management. Aging Clin Exp Res.

[9] Gil S, Jacob Filho W, Shinjo SK, Ferriolli E, Busse AL, Avelino-Silva TJ (2021). Muscle strength and muscle mass as predictors of hospital length of stay in patients with moderate to severe COVID-19: a prospective observational study. J Cachexia Sarcopenia Muscle.

[10] Kim JW, Yoon JS, Kim EJ, Hong HL, Kwon HH, Jung CY (2021). Prognostic Implication of Baseline Sarcopenia for Length of Hospital Stay and Survival in Patients With Coronavirus Disease 2019. J Gerontol A Biol Sci Med Sci.

[11] Welch C, Greig C, Masud T, Wilson D, Jackson TA (2020). COVID-19 and Acute Sarcopenia. Aging Dis.

[12] Sigfrid L, Drake TM, Pauley E, Jesudason EC, Olliaro P, Lim WS (2021). Long Covid in adults discharged from UK hospitals after Covid-19: A prospective, multicentre cohort study using the ISARIC WHO Clinical Characterisation Protocol. Lancet Reg Health Eur.

[13] Sydor W, Wizner B, Strach M, Bociąga-Jasik M, Mydel K, Olszanecka A (2021). CRACoV-HHS: an interdisciplinary project for multi-specialist hospital and non-hospital care for patients with SARS-CoV-2 infection as well hospital staff assessment for infection exposure. Folia Med Cracov.

[14] Piotrowicz K, Głuszewska A, Czesak J, Fedyk-Łukasik M, Klimek E, Sánchez-Rodríguez D (2021). SARC-F as a case-finding tool for sarcopenia according to the EWGSOP2. National validation and comparison with other diagnostic standards. Aging Clin Exp Res.

[15] Rockwood K, Song X, MacKnight C, Bergman H, Hogan DB, McDowell I (2005). A global clinical measure of fitness and frailty in elderly people. CMAJ.

[16] Clinical Spectrum. COVID-19 Treatment Guidelines https://www.covid19treatmentguidelines.nih.gov/overview/clinical-spectrum/ (Accessed 19 Apr 2022).

[17] Riesgo H, Castro A, Del Amo S, San Ceferino MJ, Izaola O, Primo D (2021). Prevalence of Risk of Malnutrition and Risk of Sarcopenia in a Reference Hospital for COVID-19: Relationship with Mortality. Ann Nutr Metab.

[18] Malmstrom TK, Miller DK, Simonsick EM, Ferrucci L, Morley JE (2016). SARC-F: a symptom score to predict persons with sarcopenia at risk for poor functional outcomes. J Cachexia Sarcopenia Muscle.

[19] Lorent N, Vande Weygaerde Y, Claeys E, Guler Caamano Fajardo I, De Vos N, De Wever W (2022). Prospective longitudinal evaluation of hospitalised COVID-19 survivors 3 and 12 months after discharge. ERJ Open Res.

[20] Levy D, Giannini M, Oulehri W, Riou M, Marcot C, Pizzimenti M (2022). Long Term Follow-Up of Sarcopenia and Malnutrition after Hospitalization for COVID-19 in Conventional or Intensive Care Units. Nutrients.

[21] Cheval B, Sieber S, Maltagliati S, Millet GP, Formánek T, Chalabaev A (2021). Muscle strength is associated with COVID-19 hospitalization in adults 50 years of age or older. J Cachexia Sarcopenia Muscle.

[22] Del Brutto OH, Mera RM, Pérez P, Recalde BY, Costa AF, Sedler MJ (2021). Hand grip strength before and after SARS-CoV-2 infection in community-dwelling older adults. J Am Geriatr Soc.

[23] Cuerda C, Sánchez López I, Gil Martínez C, Merino Viveros M, Velasco C, Cevallos Peñafiel V, et al. Impact of COVID-19 in nutritional and functional status of survivors admitted in intensive care units during the first outbreak. Preliminary results of the NUTRICOVID study. Clin Nutr. 2021;0261–5614(21)00526–4. doi:10.1016/j.clnu.2021.11.017. 10.1016/j.clnu.2021.11.017 PMC8609675 34893357

[24] Osuna-Padilla IA, Rodríguez-Moguel NC, Rodríguez-Llamazares S, Orsso CE, Prado CM, Ríos-Ayala MA, et al Low muscle mass in COVID-19 critically-ill patients: Prognostic significance and surrogate markers for assessment. Clin Nutr. 2022;S0261-5614(22)00070-X. doi: 10.1016/j.clnu.2022.02.019. 10.1016/j.clnu.2022.02.019 PMC8886683 35282986

[25] Damanti S, Cristel G, Ramirez GA, Bozzolo EP, Da Prat V, Gobbi A, et al Influence of reduced muscle mass and quality on ventilator weaning and complications during intensive care unit stay in COVID-19 patients. Clin Nutr. 2021:S0261-5614(21)00375–7. doi: 10.1016/j.clnu.2021.08.004. 10.1016/j.clnu.2021.08.004 PMC8364854 34465493

[26] Meyer H-J, Wienke A, Surov A (2022). Computed tomography-defined body composition as prognostic markers for unfavourable outcomes and in-hospital mortality in coronavirus disease 2019. J Cachexia Sarcopenia Muscle.

[27] Meyer H-J, Wienke A, Surov A (2021). Computed tomography-defined low skeletal muscle mass as a prognostic marker for short-term mortality in critically ill patients: a systematic review and meta-analysis. Nutrition.

[28] Casas-Deza D, Bernal-Monterde V, Aranda-Alonso AN, Montil-Miguel E, Julián-Gomara AB, Letona-Giménez L (2021). Age-related mortality in 61,993 confirmed COVID-19 cases over three epidemic waves in Aragon, Spain. Implications for vaccination programmes. PLoS ONE.

[29] Junior AF, Azevedo E, Paes AT, Lima E, Campos Guerra JC, Ingham SJMN (2022). Chronic diseases, chest computed tomography, and laboratory tests as predictors of severe respiratory failure and death in elderly Brazilian patients hospitalized with COVID-19: a prospective cohort study. BMC Geriatr.

[30] Sansone NMS, Boschiero MN, Ortega MM, Ribeiro IA, Peixoto AO, Mendes RT (2022). Severe Acute Respiratory Syndrome by SARS-CoV-2 Infection or Other Etiologic Agents Among Brazilian Indigenous Population: An Observational Study from the First Year of Coronavirus Disease (COVID)-19 Pandemic. Lancet Reg Health Am.

[31] Diebold M, Martinez AE, Adam K-M, Bassetti S, Osthoff M, Kassi E (2021). Temporal trends of COVID-19 related in-hospital mortality and demographics in Switzerland - a retrospective single centre cohort study. Swiss Med Wkly.

[32] Fumagalli S, Trevisan C, Del Signore S, Pelagalli G, Fumagalli C, Herbst A (2022). Atrial fibrillation and COVID-19 in older patients: how disability contributes to shape the risk profile. An analysis of the GeroCovid registry. Aging Clin Exp Res.

[33] Alves VP, Casemiro FG, de Araujo BG, Lima MA, de Oliveira S, dede Fernandes RS (2021). Factors Associated with Mortality among Elderly People in the COVID-19 Pandemic (SARS-CoV-2): A Systematic Review and Meta-Analysis. Int J Environ Res Public Health.

[34] Ortega E, Corcoy R, Gratacòs M, Cos Claramunt FX, Mata-Cases M, Puig-Treserra R (2021). Risk factors for severe outcomes in people with diabetes hospitalised for COVID-19: a cross-sectional database study. BMJ Open.

[35] Bezzini D, Schiavetti I, Manacorda T, Franzone G, Battaglia MA (2021). First Wave of COVID-19 Pandemic in Italy: Data and Evidence. Adv Exp Med Biol.

[36] Moreno-Perez O, Ribes I, Boix V, Martinez-García M, Otero-Rodriguez S, Reus S (2022). Hospitalized patients with breakthrough COVID-19: Clinical features and poor outcome predictors. Int J Infect Dis.

[37] Assis FC, Silva MCD, Geber-Júnior JC, Roschel H, Peçanha T, Drager LF (2021). Association of health vulnerability with adverse outcomes in older people with COVID-19: a prospective cohort study. Clin (Sao Paulo).

[38] Fujita K, Kashihara E, Kanai O, Hata H, Yasoda A, Odagaki T (2021). Increasing Burden of Nursing Care on the Treatment of COVID-19 Patients in the Aging Society: Analyses During the First to the Third Wave of Pandemic in Kyoto City, Japan. Front Med (Lausanne).

[39] Petermann-Rocha F, Hanlon P, Gray SR, Welsh P, Gill JMR, Foster H (2020). Comparison of two different frailty measurements and risk of hospitalisation or death from COVID-19: findings from UK Biobank. BMC Med.

[40] Rottler M, Ocskay K, Sipos Z, Görbe A, Virág M, Hegyi P (2022). Clinical Frailty Scale (CFS) indicated frailty is associated with increased in-hospital and 30-day mortality in COVID-19 patients: a systematic review and meta-analysis. Ann Intensive Care.

[41] Hewitt J, Carter B, Vilches-Moraga A, Quinn TJ, Braude P, Verduri A (2020). The effect of frailty on survival in patients with COVID-19 (COPE): a multicentre, European, observational cohort study. Lancet Public Health.

[42] Woolford SJ, D’Angelo S, Curtis EM, Parsons CM, Ward KA, Dennison EM (2020). COVID-19 and associations with frailty and multimorbidity: a prospective analysis of UK Biobank participants. Aging Clin Exp Res.

[43] Dumitrascu F, Branje KE, Hladkowicz ES, Lalu M, McIsaac DI (2021). Association of frailty with outcomes in individuals with COVID-19: A living review and meta-analysis. J Am Geriatr Soc.

[44] Corradini E, Ventura P, Ageno W, Cogliati CB, Muiesan ML, Girelli D (2021). Clinical factors associated with death in 3044 COVID-19 patients managed in internal medicine wards in Italy: results from the SIMI-COVID-19 study of the Italian Society of Internal Medicine (SIMI). Intern Emerg Med.

[45] Monserrat Villatoro J, Mejía-Abril G, Díaz García L, Zubiaur P, Jiménez González M, Fernandez Jimenez G (2022). A Case-Control of Patients with COVID-19 to Explore the Association of Previous Hospitalisation Use of Medication on the Mortality of COVID-19 Disease: A Propensity Score Matching Analysis. Pharmaceuticals (Basel).

[46] Osborne TF, Veigulis ZP, Arreola DM, Mahajan SM, Röösli E, Curtin CM (2021). Association of mortality and aspirin prescription for COVID-19 patients at the Veterans Health Administration. PLoS ONE.

[47] Salah HM, Mehta JL (2021). Meta-Analysis of the Effect of Aspirin on Mortality in COVID-19. Am J Cardiol.

[48] Hoertel N, Sánchez-Rico M, Vernet R, Beeker N, Jannot A-S, Neuraz A (2021). Association between antidepressant use and reduced risk of intubation or death in hospitalized patients with COVID-19: results from an observational study. Mol Psychiatry.

[49] Zheng W, Sun H-L, Cai H, Zhang Q, Ng CH, Xiang Y-T (2022). Antidepressants for COVID-19: A systematic review. J Affect Disord.

[50] Oskotsky T, Marić I, Tang A, Oskotsky B, Wong RJ, Aghaeepour N (2021). Mortality Risk Among Patients With COVID-19 Prescribed Selective Serotonin Reuptake Inhibitor Antidepressants. JAMA Netw Open.

[51] Lenze EJ, Mattar C, Zorumski CF, Stevens A, Schweiger J, Nicol GE (2020). Fluvoxamine vs Placebo and Clinical Deterioration in Outpatients With Symptomatic COVID-19: A Randomized Clinical Trial. JAMA.

[52] Gori M, Berzuini C, D’Elia E, Ghirardi A, Bernardinelli L, Gavazzi A (2022). Antecedent use of renin-angiotensin system inhibitors is associated with reduced mortality in elderly hypertensive Covid-19 patients. J Hypertens.

[53] McKeigue PM, Kennedy S, Weir A, Bishop J, McGurnaghan SJ, McAllister D (2021). Relation of severe COVID-19 to polypharmacy and prescribing of psychotropic drugs: the REACT-SCOT case-control study. BMC Med.

[54] Chou MY, Nishita Y, Nakagawa T, Tange C, Tomida M, Shimokata H (2019). Role of gait speed and grip strength in predicting 10-year cognitive decline among community-dwelling older people. BMC Geriatr.

[55] Hashida R, Matsuse H, Bekki M, Iwanaga S, Higuchi T, Hirakawa Y (2021). Grip Strength as a Predictor of the Functional Outcome of Hip-Fracture Patients. Kurume Med J.

